# Metabolomic Approaches to Study Chemical Exposure-Related Metabolism Alterations in Mammalian Cell Cultures

**DOI:** 10.3390/ijms21186843

**Published:** 2020-09-18

**Authors:** Aneta Balcerczyk, Christian Damblon, Bénédicte Elena-Herrmann, Baptiste Panthu, Gilles J. P. Rautureau

**Affiliations:** 1 Department of Molecular Biophysics, Faculty of Biology and Environmental Protection, University of Lodz, Pomorska 141/143, 90-236 Lodz, Poland; aneta.balcerczyk@biol.uni.lodz.pl; 2 Unité de Recherche MolSys, Faculté des sciences, Université de Liège, 4000 Liège, Belgium; c.damblon@ulg.ac.be; 3 CNRS, INSERM, IAB, Univ Grenoble Alpes, Allée des Alpes, 38000 Grenoble, France; benedicte.elena@univ-grenoble-alpes.fr; 4 CarMeN Laboratory, INSERM, INRA, INSA Lyon, Univ Lyon, Université Claude Bernard Lyon 1, 69921 Oullins CEDEX, France; baptiste.panthu@univ-lyon1.fr; 5 Hospices Civils de Lyon, Faculté de Médecine, Hôpital Lyon Sud, 69921 Oullins CEDEX, France; 6 Centre de Résonance Magnétique Nucléaire à Très Hauts Champs (CRMN FRE 2034 CNRS, UCBL, ENS Lyon), Université Claude Bernard Lyon 1, 69100 Villeurbanne, France

**Keywords:** xenobiotics, exposome, metabolomics, NMR, mass spectrometry, cell cultures, endometabolome, exometabolome

## Abstract

Biological organisms are constantly exposed to an immense repertoire of molecules that cover environmental or food-derived molecules and drugs, triggering a continuous flow of stimuli-dependent adaptations. The diversity of these chemicals as well as their concentrations contribute to the multiplicity of induced effects, including activation, stimulation, or inhibition of physiological processes and toxicity. Metabolism, as the foremost phenotype and manifestation of life, has proven to be immensely sensitive and highly adaptive to chemical stimuli. Therefore, studying the effect of endo- or xenobiotics over cellular metabolism delivers valuable knowledge to apprehend potential cellular activity of individual molecules and evaluate their acute or chronic benefits and toxicity. The development of modern metabolomics technologies such as mass spectrometry or nuclear magnetic resonance spectroscopy now offers unprecedented solutions for the rapid and efficient determination of metabolic profiles of cells and more complex biological systems. Combined with the availability of well-established cell culture techniques, these analytical methods appear perfectly suited to determine the biological activity and estimate the positive and negative effects of chemicals in a variety of cell types and models, even at hardly detectable concentrations. Metabolic phenotypes can be estimated from studying intracellular metabolites at homeostasis in vivo, while in vitro cell cultures provide additional access to metabolites exchanged with growth media. This article discusses analytical solutions available for metabolic phenotyping of cell culture metabolism as well as the general metabolomics workflow suitable for testing the biological activity of molecular compounds. We emphasize how metabolic profiling of cell supernatants and intracellular extracts can deliver valuable and complementary insights for evaluating the effects of xenobiotics on cellular metabolism. We note that the concepts and methods discussed primarily for xenobiotics exposure are widely applicable to drug testing in general, including endobiotics that cover active metabolites, nutrients, peptides and proteins, cytokines, hormones, vitamins, etc.

## 1. Introduction

At the organism or single-cell levels, biological entities are constantly affected by their environment i.e., the multiple chemical and physical stimuli inducing modulation and adaptation of physiological processes that define the “exposome” [1,2]. Adaptations that occur in response to changes in gene material and expression, transcriptional programs, or proteins and their post-translational modifications, are described by a range of modern -omics sciences. Among those, chemical and signaling processes controlled by low molecular weight compounds, either natural endogenous metabolites or xenobiotics, can be characterized by metabolomic approaches. Metabolites are defined as small compounds of less than 1.5 kDa that belong to a variety of molecular classes such as lipids, carbohydrates, amino acids, nucleotides, or organic acids, which constitute substrates, intermediates, and products of metabolic pathways [3].

Molecular-omics stand at the forefront of future medicine, and multi-omics approaches support the development of systemic molecular diagnosis, preventive medicine, and personalized treatments [4,5]. Metabolomics plays a key role in this translational omics cascade [6,7], and equally strives to address the chemical complexity of our environment. Analysis of metabolome has notably developed as a cornerstone approach for assessment of the global exposome and its interaction with human health [8]. Both, metabolomic and pharmacometabolomic studies are suitable for vast ranges of pre-clinical and fundamental studies, particularly efficient to detect the effect of xenobiotics on a large range of biological model systems [9]. Post-drug metabolomic profiles have become a valuable and important predictor of drug effectiveness and possible side effects.

### 1.1. Biological Systems Exposure to Xenobiotics

Xenobiotics are defined as chemical compounds that are foreign to an organism, i.e., that are not endogenously produced or expected to be present naturally within the organism [10]. A substance that is a xenobiotic to one organism may be natural to another. An important category of xenobiotics arises as a result of human activity, more and more along with the development of industry (synthetic xenobiotics). However, there are also natural xenobiotics that can access the human body as plant alkaloids or fungal toxins. Organisms have always been exposed to xenobiotics and have prepared themselves for such exposure by developing resistance mechanisms and/or chemical defense, i.e., xenobiotic metabolism that decreases the toxicity of many chemicals and facilitates their removal [11]. Xenobiotics can induce an immense variety of biological effects that can be observed at many levels including alterations in cellular and subcellular structures, physiology, behavior, growth, reproduction, and mortality. Xenobiotics evaluation does not rely on a binary positive/negative classification of their impact on metabolism and homeostasis, as properties may change depending on their dose/concentration, on the exposed organism, exposure time, mode of administration, or effective metabolism mechanisms. Some chemicals affect the organism to a limited extent, more locally for example cosmetics or personal care products. Others cause a systemic response by increasing the body’s endurance and helping to boost metabolism e.g., dietary supplements, or to support therapeutic processes as drugs/chemotherapeutics/antibiotics. Those classified as allergens or toxins may induce a severe inflammatory reaction leading to anaphylactic shock, overwhelming the protective immune response after either primo-exposure or sensitization [11,12]. Such a response is highly organism-dependent, as toxic compounds for a given organism, may be harmless or even essential to others.

The intrinsic definition of xenobiotics assembles together many molecules of different structures, origins, and chemical or metabolic properties, merging for example environmental and food-derived, pharmaceutical or human-produced pollutant molecules. This definition in itself raises specific and complex issues, such as the possibility to generate a multi-dimensional classification of metabolites, where metabolites can belong to different groups, compare subgroups of xenobiotics, search for similar patterns or study their interactions, or identify common metabolic features. This area is explored using bioinformatics and cheminformatics computational methods and is supported by the development of xenobiotics databases such as XMETdb (http://www.xmetdb.org) [13].

### 1.2. Evaluation of Individual Chemicals on Biological Systems

The constantly growing number of new xenobiotics, appearing with the development of industry and modern technologies, drives massive analytical needs to assess their impact on live organisms in terms of physiology modification and generation of potential side effects [14]. This is a very challenging task, given the complexity of a living individual’s biochemistry and the possibility of interaction between the xenobiotics that rarely affect biological systems separately. A combination of molecules may completely modulate the biological response of individual ones and chemical mixtures can present additive, synergistic, or antagonistic outcomes [15]. Interaction between chemicals is defined as synergistic when producing an effect greater than the sum of individual effects from compounds in the mixture. Conversely, weaker effects stem from antagonistic interactions [16,17].

**Figure 1:**
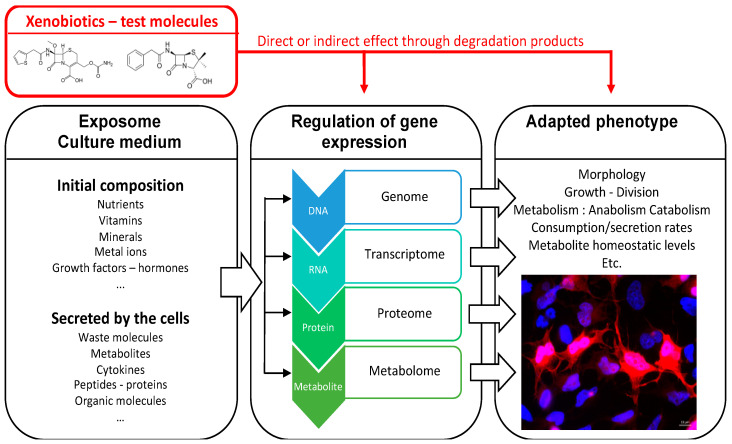
Cells are constantly exposed to chemical stimuli through their growth medium. They adapt their molecular machinery to their environment by constantly adjusting all levels of gene expression regulation. Like other metabolites, xenobiotics can act at every level of regulation from the genome to the metabolome to modulate the organism’s phenotype, underlying the translational value of -omics studies. The study of metabolite levels is a sensitive indicator of xenobiotics biological activities.

Understanding the activity of xenobiotics over biological systems, but also their interactions, and revealing their mechanisms of action is very important, especially in pharmacotherapy, including combined treatments, or toxicology [18]. The recent development of metabolomic techniques, based on high-throughput analytical platforms and sophisticated data analysis software, opens a new window for detection, identification, and quantification of hundreds of chemical compounds simultaneously [9,19], either at a baseline or in response to single or multiple exposures (Figure 1).

### 1.3. Cellular Metabotypes: The Stepping-Stones towards Organisms Phenotypes

Metabolic modeling exploits multiple facets of metabolomics approaches for the characterization of whole organisms, their pathologies, and adaptations to changeable environmental conditions. Metabolomic analyses of body fluids refer to systemic overviews of an organism’s state, however concealing important information about the behavior of cells that underlie changes in the dynamic homeostasis at the organism level [9,20]. Modeling cellular metabolism allows one to investigate biological processes but also serves a predictive role for verifying diagnostic hypotheses using cell cultures in vitro. The benefits of using cell culture models in metabolomics studies are multiple and include low experimental costs and high data reproducibility. They allow the determination of metabolite biomarkers that characterize physiological and pathological states of the cell and importantly provide data easier to interpret than those obtained in animal models or human biofluids [20]. Cell culture also offers a large panel of options regarding the choice of tissue-specific derived cells, allowing to target cell-type-specific responses [12]. Recent developments in metabolomics technologies take advantage of cell culture profiling to extrapolate the results to whole systems [5,21]. So far, metabolomic analyses have been used to identify the biochemical response of cells to xenobiotics in the context of pharmaco-toxicological studies [12,22,23,24], to characterize bioproduction [25,26], cell plasticity and carcinogenesis [27], and environmental [28,29] or nutritional interventions [30,31].

In this review, we summarize current achievements in cell culture metabolome analyses, with particular focus on analytical techniques, especially opportunities given by nuclear magnetic resonance spectroscopy (NMR) and mass spectrometry (MS) and their contribution to metabolic profiling of biological samples, indicating the significance of cell models for the analysis xenobiotics-induced biological effects.

## 2. Metabolic Phenotyping of Cell Culture Metabolism: Analytical Strategies and Platforms

Comprehensive metabolic profiling requires high-resolution analytical techniques that should ideally be able to profile thousands of small molecules of highly variable physical and chemical properties [32], with dynamic concentration ranges distributed over six to nine orders of magnitude [33]. While stunning technological progress has been achieved over the last decades [34], modern analytical platforms still cannot identify and quantify the entire metabolome of a biological system/sample [35]. To date, NMR spectroscopy and mass spectrometry largely dominate the field of metabolomics [34,36]. The two platforms rely on very different physical and chemical strategies to generate high-resolution metabolic profiles from biological samples. Interestingly, corresponding outputs only partially overlap in terms of detected metabolites, and both technologies that offer complementary advantages and disadvantages [37] have proven to deliver robust results for metabolomics studies. Recent advances tend to associate MS and NMR through integrated studies to maximize the sensitivity and precision of metabolomics studies [34,38,39]. The information-rich datasets generated from NMR or MS are subjected to computational and statistical analyses to identify event-related metabolite changes and potential biomarkers [40]. The signal processing, assignment, and data mining processes are key components of metabolomics studies that have seen tremendous progress over the last decade thanks to the development of computational methods and databases [34]. This section aims to guide the choice of a suitable analytical technique and present the central concepts sustaining metabolomics workflows in the context of cell culture studies, from metabolite extraction to statistical analyses. While this section mostly focuses on the analytical metabolomics workflow, Table 1 provides a complementary overview of broader experimental parameters that should be considered for experimental design and multiple aspects that may guide their choice.

### 2.1. Analytical Strategies

#### 2.1.1. Targeted and Untargeted Metabolomics Approaches

Two broad options can be considered that concern metabolomic investigations that are both suited to the study of cell cultures: targeted or untargeted approaches [43]. They differ conceptually and analytically, but the two of them rely on the acquisition of quantitative data, relative abundance, or even simple detection of metabolites [65]. Targeted approaches focus on a set of predefined metabolites to be measured in biological samples, such as metabolites from a given pathway, or belonging to a certain class of molecules (e.g., fatty acids). Targeted metabolomics relies on hypotheses or preliminary data on the mode of action of a perturbator, and have proven useful for assessing the response of biological organisms to environmental xenobiotic exposure [66,67]. On the other hand, untargeted metabolomics studies capture more comprehensive pictures of biological systems by collecting all possible data available from a given technique, without any a priori on the compounds detected. Untargeted analytical approaches provide access to hundreds or thousands of metabolite signals [34], and sophisticated statistical methods are required to extract and identify relevant metabolite alterations and biomarkers associated with chemical exposure [60,68].

#### 2.1.2. Isotope Tracing and Fluxomics

As life is built upon carbon chemistry, carbon-13 is a stable isotope tracer frequently used in NMR and MS metabolomics studies [69,70,71]. In NMR, individual 13C atoms can be tracked through isotopomers, i.e., isomers that differ only by the position of 13C, and followed over chemical transformations of metabolic pathways. MS cannot distinguish isotopomers but can determine accurately isotopologs, i.e., the number of 13C atoms integrated into metabolites as well as the level of enrichment. In fluxomics experiments, culture cells are put in contact with 13C-labelled substrates, such as glucose, and 13C labeled product metabolites are quantified either by NMR or MS. This permits a very precise quantification of fluxes of matter along metabolic pathways [70,72]. Using this approach, direct impacts of a perturbation, be it a chemical added to the cell culture or a genetic mutation, can be identified. Fluxomics measurements are usually carried out after a fixed delay, but multiple time point measurements can also be conducted to obtain time-resolved results [70].

### 2.2. NMR and MS Technologies for Metabolomics

Though metabolomics is conceptually independent of any particular analytical method, two analytical techniques dominate the field: nuclear magnetic resonance [73,74,75] and mass spectrometry [34,76]. Both have been extensively developed, applied, and validated in the context of metabolomics studies [77].

#### 2.2.1. NMR Spectroscopy

NMR is a fast and highly reproducible analytical technique that is based on the interaction between a nuclei spin and an external magnetic field. High magnetic fields generate the strongest nuclei polarization levels, which translates into optimal NMR sensitivity and resolution. NMR spectroscopy at 600 MHz (1H resonance frequency) is currently considered as the gold-standard for biomedical metabolomics research, offering adequate and cost-effective analytical performance, with broad access to the scientific community [73,74]. NMR is a non-destructive technique, where samples are fully recovered after analysis. Its conventional setting in solution requires minimal pre-analytical sample processing [51], with no chemical derivatization, and is a robust quantitative technique that offers excellent repeatability and reproducibility across large numbers of samples, NMR spectrometers, and NMR facilities [78]. Typical NMR analyses for metabolic profiling are fast (5 to 30 min per sample) and cost-effective. The main limitation of NMR is its low intrinsic sensitivity, with a detection limit in the micromolar range for metabolomics applications. Typically, NMR can detect in the order of between 100 metabolites in cell extracts derived from a few hundred thousand to a few millions of eukaryotic cells (typical amount for 5 mm NMR tube is a 10 cm culture dish at 80% of confluency), and identify and quantify 50 to 70 of them in cell extracts and culture media [49,52].

NMR is also a flexible technique that has long been developed to study cell metabolism under multiple settings, such as flow-probe perfusion systems or miniaturized bioreactors inside the NMR spectrometers that allow profiling cell metabolism in real-time [79]. Whole-cell pellets or intact biological tissues can also be investigated using the HR-MAS (high-resolution magic-angle spinning) NMR technique, avoiding bias induced by extraction protocols [80].

The nuclei observed by NMR and compatible with metabolomics NMR studies are spin 1/2 isotopes present at various natural isotopic abundance in biological material: 1H (proton), 13C, 15N, and 31P [74]. Protons are the most common NMR observables, due to their relatively high sensitivity, 99.98% natural abundance, and omnipresence in organic compounds. As a consequence, one-dimensional (1D) 1H NMR spectra sustain most NMR metabolomics studies. Carbon-13 NMR is impractical for high-throughput metabolomics NMR analyses due to the low natural abundance (1.1%) of carbon-13 isotopes in biological material [81]. Phosphorus-31 NMR can be exploited to study intracellular energy states, yet 31P signals from phosphorylated compounds shift heavily upon sample conditions, making signal assignment tedious [82]. Due to an intrinsic lower sensitivity and scarce natural abundance, nitrogen-15 studies rely on isotopic labeling schemes for targeted pathways analysis.

NMR signals, through their spectral position (chemical shift) and multiplicity, carry a vast amount of structural information that is exploited for metabolite annotation, and structure determination of unknown compounds in complex matrices [83]. NMR acquisition relies on a variety of radio-frequency pulse schemes to provide high-throughput detection of 1D metabolic profiles [75,84], which either allow absolute quantification of metabolites (1H NOESY scheme) or relative quantification when using spectral editing schemes (e.g., 1H CPMG experiment) [74,85,86]. Two-dimensional (2D) NMR acquisition schemes that require long experimental times (a few hours to days) are typically recorded on a representative sample for metabolite annotation purposes. The routine assignment strategy relies on a set of 1D and 2D correlation spectra to determine metabolites’ 1H (and 13C) shifts and compared them with spectral reference databases (see Section 2.3.2).

NMR is universally quantitative, which means metabolites concentrations can be determined from signal integration using a single reference (e.g., lactate) of known concentrations [87]. Metabolites concentrations are routinely determined from 1H 1D NOESY spectra, either automatically or interactively, by the integration of well-resolved peaks belonging to a metabolite, or using more advanced curve-fitting dedicated software or algorithms [60,73,88]. Using more advanced spectral editing NMR techniques, metabolite peaks intensities depend on the specific properties for each metabolite, such as relaxation times, and relative metabolite concentrations are obtained following equivalent procedures [86].

#### 2.2.2. Mass Spectrometry

Mass spectrometry is a sensitive and very high-resolution analytical technique that measures mass-to-charge ratios (m/z) of ionized molecules. Mass spectrometry has been a very active research field since the 1980s and a wide range of instruments and approaches have been developed and adapted for metabolomics [89]. MS now offers diverse solutions for metabolomics profiling as various separation techniques, ionization, and mass analyzer methods are combined to characterize complex mixtures of metabolites and improve analysis and identification [76,90,91].

MS is generally preceded by a separation step which aims to simplify the sample complexity and aid the mass spectra analysis. Significant advances have occurred in separation-based MS techniques, and liquid and gas chromatography (LC and GC, respectively), or capillary electrophoresis (CE) are the most frequently used separation techniques. GC has been widely used for metabolomics [92] and is most suited for the targeted analysis of volatile compounds, from original or derivatized samples [93]. Most recently, liquid chromatography and ultra-high-performance liquid chromatography (UHPLC) techniques have been developed to provide high-resolution and high-throughput capacities, with the advantage that metabolites are separated in the liquid phase, which makes derivatization unnecessary and less prone to introduce bias and artifacts [76]. LC-MS coupled to electrospray as an ionization source can now routinely identify and estimate levels of a few hundred metabolites within a single extract [94,95]. On the other side, multiple separation-free MS techniques have been successfully applied to metabolomics with direct infusion-mass spectrometry and matrix-assisted laser desorption ionization (MALDI) mass spectrometry being applicable to cell culture studies. While these techniques benefit from fewer analytical steps, they suffer from an enhanced complexity in the ions’ annotation and identification [76].

Identification of metabolites from dense and information-rich mass spectra is an ongoing challenge in MS, but remarkable progress has been made over the past decade with the development of hardware strategies, mass databases, and semi-automated or automated computational tools integrated into specialized software [76]. MS signal annotation is mostly based either on comparisons with libraries of existing experimental or theoretical data (see Section 2.3.2) or requires additional experimental data acquisition, such as tandem MS-MS or MS-MS-MS spectra, where further fragmentation of a parent ion provides additional structural information for metabolite identification [76,96,97]. Yet, a large proportion of MS signals remain unidentified. Recent strategies combining NMR and MS show a strong potential for the identification of the unknowns [38,98].

MS can deliver qualitative or quantitative data in the form of tables of metabolites detected in the complex samples [90]. However, the accuracy and reproducibility of the quantitative results are usually recognized to be a weakness as mass spectrometers tend to drift over time, and MS analyses require extensive quality control monitoring. All steps are known to be susceptible to time-variations from the pre-separation steps to the ionization and ion separation steps. Metabolite standards or spiking experiments for each metabolite can be used to estimate concentrations, while relative metabolite concentrations are usually determined from peak intensities comparison.

Generally, the pros of MS include a very high sensitivity for very dilute metabolites and very high resolution of sub-unit atomic mass differences, which allows MS to identify many more metabolites than NMR while requiring lower amounts of the sample [37]. This claim is mitigated by the actual diversity of MS instruments and experimental setups, each having dedicated applications and properties. MS is a destructive technique, i.e., samples cannot be recovered after analysis and requires extensive sample matrix optimization or derivatization. 

#### 2.2.3. A Rationale to Select the Optimal Analytical Technique?

Whether the strategy is to carry out targeted or untargeted analysis, both NMR and MS are adequate to detect and identify metabolite perturbations both qualitatively and quantitatively. Both techniques have their strengths and weaknesses and can deliver largely non-overlapping information from the same metabolomic samples [34,37]. There seemingly exists no universal answer to the question of the choice of an optimum analytical setup in the context of chemical testing on cell culture. Primarily, this choice should depend on the specific focus of the research study, but will importantly consider the accessibility of analytical platforms and availability of technological and methodological expertise for metabolomics studies. Including both techniques is an option that may yield the most comprehensive results but requires major resources and specific expertise to integrate datasets [39]. NMR is particularly stable and reproducible and is well adapted to evaluate the broad impact of perturbators over cellular metabolism by identifying small variations of mostly common metabolites. NMR is also an excellent tool to discriminate and pre-evaluate metabolic profiles before further detailed MS analyses. NMR results are quantitative and scalable and can be integrated with other analytical methods. MS strategies offer wider coverage of the metabolome and the ability to assess very low concentration metabolites and are perfectly adapted to identify low concentrated biomarkers [76], although they require more extensive setup optimization and lead to a higher complexity of data analysis.

### 2.3. Evaluating the Impact of Xenobiotics on Cell Cultures: A Metabolomics Workflow

**Figure 2:**
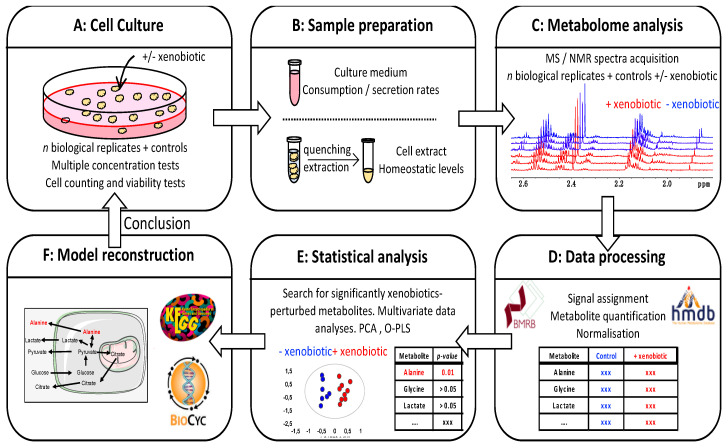
Detection of xenobiotics-induced metabolism alterations in mammalian cell cultures: general metabolomics workflow. (**A**) Cell cultures are performed in the presence of a concentration range of xenobiotic. Adequate controls are used: dishes without cells to determine consumption/secretion rates, dishes for cell counting, as well as biological parameters determination such as viability tests. (**B**) Metabolomics sample preparation: the culture medium that contains extracellular metabolites is isolated by centrifugation; the cell pellet is subjected to metabolism quenching and metabolite extraction protocols to extract the intracellular metabolites. (**C**) The metabolome analysis by MS and/or NMR data acquisition allows for the obtaining of rich experimental data. (**D**) Data processing, metabolite identification, quantification, and normalization of the results. (**E**) Statistical analyses to identify metabolites levels that are altered as a consequence of xenobiotic testing (this step aims to identify individual biomarkers as well as complex metabolites signatures). (**F**) Model reconstruction and integration of the results over metabolic pathways.

From an analytical point of view, the typical metabolomics workflow for the analysis of cell material can be divided into three consecutive steps: metabolite extraction and sample preparation, NMR or MS analysis to collect experimental metabolic profiles and data analysis including statistics to identify the main features associated with a biological perturbator (Figure 2) [99]. Unlike other omics approaches, the metabolome is highly chemically complex, dynamic, and sensitive to both biological and analytical conditions. As a consequence, all steps along the full procedure are prone to introduce bias and artifacts that will impact the biological conclusions. Experimental design must be thought through carefully and suitable standardized protocols for sample preparation should be adopted with proper training of the biologist to ensure smooth and reproducible execution. The same batches of chemicals and consumables are to be used for all samples, and sample handling should be carefully randomized across different sample classes (for the date of sample preparation, sample collection, chemical analysis, etc.) [100,101].

#### 2.3.1. Metabolites Extraction

The extraction of metabolites from biological samples is central to metabolomics studies. Cell cultures can generate two categories of metabolomic information: intra- and extra-cellular metabolic profiles. The obtention of samples from extracellular media is straightforward and requires a single centrifugation step of culture supernatants to remove cells and debris. On the contrary, numerous protocols aimed at the extraction of metabolites from biological matrices have been developed for NMR and MS analyses, some of which have been specifically tuned for cell cultures [49,51,52,56,102,103,104,105,106,107,108,109,110]. These protocols are quite similar in principle for both analytical platforms but suit individual requirements such as avoiding high concentrations of protonated solvent molecules as concerns NMR profiling. NMR tolerates a certain amount of macromolecules in the sample tube, though a lesser amount of proteins, lipids, and nucleic acid generate sharper peaks and easier spectral interpretation. Critical steps towards intracellular metabolites extraction include initial washing of the cells to remove extracellular contaminants while minimizing the delay before metabolism quenching. A complete arrest of metabolism and enzymatic activity as quickly as possible after culture medium removal is essential for meaningful profiling of the intracellular metabolome [52].

The existence of numerous metabolite extraction protocols stems from the fact that metabolites possess a broad diversity of physical and chemical properties that influence their extraction yields. Hence, fit-for-all protocols result from experimental compromises, and protocols can be fine-tuned to optimize specific extraction conditions for different classes of metabolites [49]. Beyond differences in metabolites extraction yields, the ergonomics and likely associated reproducibility of protocols, as well as the use of toxic reagents, are also key points to consider, especially when a large number of cell cultures are involved [52]. Most metabolomic extraction protocols eventually target subsets of metabolites, such as water-soluble metabolites or lipids. Various strategies have been optimized using a one-phase liquid extraction, which is usually sufficient to remove most macromolecules such as proteins or nucleic acids [49,52]. Polar metabolites are mainly extracted using methanol or acetonitrile-based solutions followed by centrifugation to precipitate macromolecules and hydrophobic metabolites such as lipids. Biphasic extraction protocols, generally based on a mixture of chloroform, water, and methanol, are also employed for the simultaneous extraction of hydrophilic and lipophilic metabolites into two separate phases [51].

Metabolic profiles may also be impacted by metabolites stability in various conditions during processing, storage, and analysis, such as drying state, storage temperature (freezing), experimental temperature, pH, and buffer conditions, but also by potential chemical cross-reactions between metabolites or oxidation processes, etc. [111,112]. Thus, it appears critical to standardize every step of the protocol and randomize sample handling to minimize biologically irrelevant bias related to sample processing [113]. Under standardized conditions, reliable and reproducible results can be obtained [100,114]. Cell supernatants and extracts are typically stable for a few hours on ice or at 4 °C, weeks at −20 °C and months at −80 °C [115]. 

#### 2.3.2. Metabolites Identification and Quantification

Once NMR or MS spectra have been acquired, metabolite identification is a crucial step, and the current limitation to many metabolomic studies. Detailed metabolite annotation strategies are specific to the analytical techniques and instrumental setup. Metabolite assignment is generally obtained by the comparison of peak features (such as multiplicity, coupling constants, and chemical shifts for NMR, and m/z ratios, isotope pattern, element composition, fragmentation and/or retention time for (LC-) MS) against public, commercial or in-house spectral libraries of known metabolites [60,116,117,118].

The main difficulty lies in the immense repertoire of metabolites in the order of tens to hundreds of thousands for mammalian culture cells, which largely exceeds the size of current databases and the numerous ambiguities for chemical identification [113,119]. Moreover, the use of databases to identify metabolites is specifically limited in the case of unknown molecules, such as most xenobiotics and xenobiotics-derived metabolites. A rigorous approach to both identification and quantification of metabolites in a complex sample remains the use of standards, for example using spiking in NMR [83] or MS experiments [120]. In the latter case, spiking experiments often rely on isotopically labeled compounds [92]. Globally, due to its higher sensitivity and resolution, MS can identify many more metabolites than NMR [121].

Metabolite quantification is essential to biological interpretations and modeling of metabolomic data [34] and has been a focus of many methods’ development [33,85,86,90,122]. As concerns cellular profiles, normalization of the data is essential. Intracellular metabolite quantities are usually normalized to cell number, yet the protein or DNA content can also be used as an alternative [56]. As the exometabolomes are often interpreted in the form of consumption/production rates, normalization should take into account the growth and division of the cells over the experiment time. To count cells, at least one extra seeded culture dish should be added to the experiment for each group [123]. If the addition of a xenobiotic significantly perturbs the growth capacity of a cell culture, using smaller volumes of culture media, to accentuate metabolites variations, and collecting supernatants over shorter periods can be utilized to shorten cell cultures, moderate normalization issues, and facilitate comparison with controls [47].

#### 2.3.3. Multivariate Data Analyses and Model Construction

Both NMR- and MS-based metabolomics studies generate large amounts of data, from which important features that are associated with a perturbator must be identified among hundreds or thousands of spectral variables. Such data reduction is traditionally achieved using multivariate statistical analyses that include unsupervised and supervised approaches [50,124]. Unsupervised approaches, which mainly consist of principal component analysis (PCA) and hierarchical clustering analysis (HCA), examine the global variance over a dataset without knowledge of any class membership. This type of approach is often conducted in the early stages of data analysis and used to check the homogeneity of the dataset, identify the main sources of variation, and pinpoint outlier samples. When the biological effect of the perturbator is strong, it may be readily identified at this early step. Further supervised techniques, such as orthogonal projection onto latent structures (O-PLS) discriminant analysis, exploit information of samples class membership to optimize their discrimination. Such enhanced features detection implies higher risks of overfitting the statistical models, and requires careful use of validation methods [59,124,125]. Multivariate data analyses (MVDA) can be employed to examine either unassigned spectral raw datasets or tables of relative or absolute metabolite concentrations. The use of unassigned spectral data tends to offer the highest discrimination potential as the full spectral fingerprint contributes to the statistical model. Yet, discriminatory spectral variables can remain challenging to annotate [36]. On the other hand, MVDA from metabolite concentration tables tend to generate more reliable results, but miss the discrimination potential of potentially important unassigned signals [124]. Although MVDA are validated and powerful statistical tools that have delivered solid results in the field of metabolomics, new algorithms and statistical approaches emerge intending to further improve group separation and reconstruct metabolic models. These exciting methods include machine learning developments such as random forest, support vector machines (SVM), and self-organizing map (SOM) algorithms [126].

Despite the distribution and availability of free or commercial computational solutions and user-friendly software, the statistical analyses of metabolomics datasets remain a critical step in the analytic workflow. No statistical method can yet pretend to offer a universal solution to the metabolomics investigations and work for every dataset [127]. Indeed, over the intrinsic difficulty of identifying weak correlations between metabolites and xenobiotic-induced perturbations, all computation methods rely on different intrinsic hypotheses. Each method displays unique pros and cons, and require method-specific fine data manipulations and handling, such as normalization or weighting. We emphasize that these manipulations, yet essential, are prone to produce highly variable and possibly false results, artifacts or model overfit, and require dedicated validation. For example, multivariate data analyses are built on linear regression models that hypothesize linearity between the level of xenobiotic-induced perturbation and the biological response, which may not be verified in all cases [128]. As a consequence, we strongly recommend that statistical analyses are always conducted under the supervision or validation of experts in statistics, even when conducted on user-friendly data analysis platforms.

Once significant metabolites features are identified, most studies aim to understand the underlying biological mechanisms and conceptualize a mode of action of the perturbator upon metabolic pathways [32]. This is a challenge as the problem is intrinsically mathematically under-determined (too little data are available to reconstruct a comprehensive or even simplified metabolic model). A single modification of an impacted metabolite at homeostasis in the cell can be explained by multiple metabolic hypotheses, such as upstream or downstream modification of a metabolic enzymatic reaction [129,130]. Besides, many complex sensing systems are constantly regulating cellular metabolism at every level, from gene expression to protein activity to compensate for a dysregulation [9,131]. However, many exciting computational tools progress toward this goal to identity mechanistic models of perturbation, for example by mapping graphically the impacted metabolites onto metabolic pathways or by using elaborate strategies that integrate various levels of experimental and theoretical data [40,132,133,134,135,136]. These rapidly developing approaches can integrate metabolite enrichment estimation on metabolic pathways maps (integrated into databases such as the Kyoto Encyclopedia of Genes and Genomes (KEGG)) but also data fusion with other -omics datasets and reconstruction of complete metabolic networks based on genome-scale data from transcriptomic analysis [137,138,139].

## 3. Addressing Cellular Response to Xenobiotics

### 3.1. Complementary Insights from Intra and Extracellular Metabolomes

Cells continuously sense the extracellular environment composition and adjust their metabolic machinery to sustain adequate anabolic, catabolic, and energy levels [46]. Such regulation of the metabolic machinery, through enzymatic activities and metabolic pathways adaptations, leads to global metabolism modifications and new homeostatic states [140]. The choice of culture conditions is then not only essential to portray a cell-intrinsic metabolism profile [46] but also to reveal xenobiotic-induced deregulations. Sometimes, it can even be useful to compare the metabolic response of cells placed in nutrient-controlled conditions to evidence a metabolic phenotype [47]. Chemical-induced metabolism perturbations appear as alterations of intracellular metabolite levels at homeostasis, but also as modifications of matter fluxes through enzymatic reactions and metabolic pathways; such fluxes are usually determined using stable isotope tracer integration by fluxomics-type experiments [141] but also by simple measurements of consumption/secretions of metabolites into the cell culture medium [47,142,143]. Characterizing both the intra- and extracellular metabolites thus offers complementary information to generate detailed metabolic models.

In the context of cell cultures, intracellular metabolites are most frequently profiled for cells cultured in stable conditions and allowed to adapt to the media before metabolism quenching and profiling [52]. Metabolite levels measured report homeostatic conditions and constitute a snapshot of intracellular metabolic activities. Many tools and computational strategies are available to aid interpreting intracellular xenobiotics-induced perturbations, making intracellular profiling of cell cultures a central tool to determine the effect of xenobiotics on simplified biological systems. Extracellular metabolites, in the case of cell cultures, correspond to metabolites exchanged between the cells and their environment. Characterization of cell culture media is eventually less common than the profiling of intracellular metabolites. However, the quantitative measurement of exometabolites in supernatants after cell culture, and comparison to control media not put in contact with cells, allows the determination of consumption and secretion rates per unit of time that convey rich insights over cellular metabolism. Indeed, exometabolites rates are excellent reporters of the cell physiology that allow for the predicting of the intracellular metabolic state and evidence mechanisms of chemical-induced metabolic reprogramming [20,144]. For example, glucose consumption and lactate/pyruvate secretion rates are simple to determine yet powerful indicators of the glycolytic activity in cells [47], and the extracellular secretion of citrate and succinate evidence mitochondrial metabolism remodeling. Both activities may be less straightforwardly monitored from intracellular metabolite profiles that are subjected to homeostatic regulation [47]. Complementing intracellular profiles with extracellular metabolites data provides deeper insights of xenobiotic-induced perturbations of the cellular metabolic physiology. Moreover, contrary to the intracellular content, cell supernatants are abundant and do not require extensive extraction protocols. They are uniquely malleable to experimental conditions as the intensity of the biological response is both dependent on the duration of the culture (longer cultures generate larger effects on metabolite concentrations), and the volume of the culture medium for the same number of cells (that affects the extracellular metabolites pool size). These options are precious when working with low abundance or difficult to expand cell cultures, such as stem cells, as they allow to amplify the amplitude of biological effects [47,143].

### 3.2. Identification of Specific Xenobiotic Metabolism

Great care should be taken when defining in vitro conditions for chemical testing to ensure the relevance of the final results. Indeed, xenobiotics molecules can be susceptible to chemical degradation or modifications that may deeply affect their activity or bioavailability. Sample conditions such as temperature, pH, redox status, and ionic strength may strongly modulate this activity either directly, for example by shifting redox equilibria, keto-enol balance, or the speciation of metal ions [63], or indirectly via a global control and reprogramming of metabolism and gene expression [145]. The stability of chemical structures should also be taken into account. Indeed, many spontaneous reactions may occur such as the glutamine to pyroglutamate conversion, or amino-acids oxidation and deamidation. All these reactions depend on chemical concentrations and mixture composition and may occur at every step of the metabolomics workflow. This is the reason why we recommend running cell-culture controls with medium only and without cells, rather than using the initial culture medium as a reference to determine the consumption/production rates. In vivo, the situation is even more complex as numerous xenobiotic biological effects are caused by their biotransformation products rather than the parent compound [146].

Organisms have developed protection systems to neutralize potentially harmful molecules from their environment. Xenobiotic metabolization processes can be evidenced in cell cultures by observing the appearance of xenobiotic-derived metabolites [147]. Xenobiotic-derived metabolites are usually difficult to identify as they are: (i) most often low concentrated (xenobiotics are usually tested at low concentration, and metabolic intermediates are transient and low populated), (ii) often chemical structures absent from reference databases, and (iii) few among thousands of metabolites issued from the regular cell metabolism. A typical application of 13C labeling is the follow-up of metabolization of a 13C-labeled chemical such as a xenobiotic [148,149]. Both intracellular and extracellular metabolites provide complimentary pictures of xenobiotics metabolization processes. Mass spectrometry is mostly used in this situation for its excellent sensitivity and resolution and mixtures of 13C-labeled and unlabeled compounds are often employed for metabolite identification and comparative quantification. Mass peaks of labeled metabolites will appear with a known mass difference as compared to unlabeled ones [90]. This strategy is often aided by MS software, such as MetExtract II [150]. NMR is sometimes used to identify new chemical structures but less adapted to follow the fate of xenobiotics in cell cultures since the detection limit can be higher than the concentration of xenobiotics added to the cell cultures when testing their biological activities.

### 3.3. Multi-Omics Studies of Xenobiotics Impact on Cell Cultures

Exposing cells to xenobiotics, and more generally to modifications of their chemical environment triggers all biological regulatory systems and impact all types of biomolecules. This explains why -omics approaches, essentially metabolomics, proteomics, transcriptomics, genomics, and epigenomics, have become central to all facets of life science. The opportunity to operate multi-omics studies to capture the full dynamic range of a biological response is an unprecedented opening of research, recently enabled by the development of adequate analytical, computational, and visualization solutions and strategies [151,152,153]. Multiple investigations, including cell culture studies of xenobiotics, demonstrate that the integration of multiple -omics leads to a considerable increase of sensitivity to detect xenobiotics effects on biological systems. They also open doors to a complete identification of molecular mechanisms of biological adaptations, providing a systemic understanding of xenobiotics-induced perturbations or toxicity pathways [154,155,156,157].

## 4. Conclusions

Metabolomics has immensely progressed over the last decade, with developments of analytical methods and computational solutions, to offer numerous applications in systems biology for medical research, disease diagnostic, or personalized medicine. However, many challenges remain in experimental methodology to characterize the full complexity of biological samples while diminishing sample-to-sample variability. Much research is active in the field of mass spectrometry and concern all aspects of the analysis, from the pre-separation, to ionization, ion separation, and detection steps and too many approaches are being explored to be discussed here. As concerns NMR, dissolution dynamic nuclear polarization (dDNP) opens exciting future perspectives for high-sensitivity NMR metabolomic detection [158,159].

Metabolomics studies of cell cultures and measurement of intra- and extracellular metabolites constitute ideal tools to evaluate both the effect of a chemical over the central cellular metabolic pathways, but also its potential toxicity or degradation, in an accessible, manageable, and practical biological setup. Importantly, such non-invasive approaches are perfectly applicable using a variety of cell lines and scalable from a unique molecule testing to high-throughput screening of biological, pharmaceutical, environmental molecules, be they endo- or xenobiotics, drugs, proteins, metabolites, etc. Metabolomics of cell culture offers unique opportunities to characterize the effect of chemicals over broad ranges of biological systems.

As metabolites represent the final expression of all levels of genomic, transcriptomic, and proteomic regulations, their measurement offers a fantastic snapshot of cellular physiology. Multiple well-validated methods in metabolomics, from sample preparation, NMR and MS analytical solutions, data processing, statistical analysis, and model reconstruction techniques allow to efficiently identify the effect of xenobiotics and chemicals over metabolic and cellular functions and their associated biomarkers. Ongoing developments concerning all these steps constantly open new possibilities, with more metabolites profiled, better precision and accuracy, and integration into more precise biological models.

The integration of analytical methods such as MS and NMR should be promoted to potentiate their individual strengths. This strategy is rendered accessible not only by the compatibility of sample preparation protocols for both methods but also by the development of integrated analytical platforms. Integrating both NMR and MS results allows for the coverage of a wider range of the metabolome. Cell cultures are also appropriate for multi-omics investigations (genomics, transcriptomics, proteomics, and metabolomics), and the development of high-throughput automation solutions adapted to most approaches, from cell culture to the final integration of results, enables such multi-omics studies. However, we emphasize that beyond the analytical pipelines that rely on well-trained experts to run analyses, handle datasets and computational methods, and integrate results from multiple sources, the achievement of fine metabolomics characterization relies on a deep understanding of metabolism and broad biological processes, and their intricate regulations and adaptations. Such complementary expertise is key to the success of metabolomics investigations, but is most frequently limiting their current impact.

## Figures and Tables

**Table 1:** Key aspects to consider for the experimental design and recommended bibliographic references.

Experimental Parameters/Degrees of Freedom	References
Type of samples (primary cells, cell lines; cell extracts, cell culture media)	[21,41,42,43,44,45]
Types of controls (raw medium, empty cultures, unexposed cells)	[46,47]
Xenobiotics dose and duration of the experiment	[42,48]
Single point or time series	[43,44]
Sample size/volume and number of replicates	[49,50]
Growth conditions (medium, temperature, normoxia/hypoxia)	[41,45]
Collection and/or extraction protocols (collection method, solvent mixture, quenching)	[49,51,52,53]
Normalization method (sample weight/volume, cell count, total spectral integral)	[54,55,56]
Analytical technique (MS, NMR)	(this review)
Use of stable isotope labels/tracers	[57,58]
Data analysis/statistical software	[59,60]
**Key Aspects to Consider**	
Objectives of the study (identification of targeted pathway alterations, global metabolism modifications, biomarkers)	[21,61]
Mode of action hypotheses	[43,62]
Kinetics of xenobiotics action, cell viability	[46]
Availability of standard operating procedures, optimized protocols	[44,53]
Xenobiotics chemical properties (solubility, stability)	[63]
Heterogeneity of the samples/inter-individual variability (in vivo/in vitro, degree of infection, degree of gene inactivation/overexpression)	[44,64]

## Abbreviations

BMRB	BioMagResBank
CE	Capillary electrophoresis
GC	Gas chromatography
HCA	Hierarchical cluster analysis
HMDB	Human metabolome database
KEGG	Kyoto Encyclopedia of Genes and Genomes
LC	Liquid chromatography
MALDI	Matrix-assisted laser desorption ionization
MS	Mass spectrometry
MVDA	Multivariate data analyses
NMR	Nuclear magnetic resonance
O-PLS	Orthogonal projection onto latent structures
PCA	Principal component analysis
SOM	Self-organizing map
SVM	Support vector machines
UHPLC	Ultra-high-performance liquid chromatography
